# Supervised Nasal Saline Irrigations in Otitis-Prone Children

**DOI:** 10.3389/fped.2019.00218

**Published:** 2019-05-31

**Authors:** Sara Torretta, Lorenzo Pignataro, Tullio Ibba, Francesco Folino, Miriam Fattizzo, Paola Marchisio

**Affiliations:** ^1^Fondazione IRCCS Ca' Granda Ospedale Maggiore Policlinico, Milan, Italy; ^2^Department of Clinical Sciences and Community Health, University of Milan, Milan, Italy; ^3^Department of Pathophysiology and Transplantation, University of Milan, Milan, Italy

**Keywords:** acute otitis media, nasal saline irrigations, children, otitis-prone children, otolaryngology

## Abstract

**Objectives:** To retrospectively investigate the impact of supervised daily nasal saline irrigations (NSI) with 0. 9% saline solution in children with a history of recurrent acute otitis media (RAOM).

**Methods:** A retrospective pilot study was planned to evaluate the possible effect of supervised NSI in reducing the number of acute otitis media (AOM) episodes in otitis-prone children aged 1–5 years, compared to children not instructed to correct NSI performance.

**Results:** Analysis was based on the data contained in 173 charts (57.3% males, mean age of 30.9 ± 7.3 months). 52.0% of children had not been instructed to perform NSI, while the remaining (48.0%) patients had received supervised NSI. At the 4-months follow-up visit a significant reduced number of AOM episodes (1.03 ± 0.14 vs. 2.08 ± 0.16; *p* < 0.001) as well as antibiotic treatments (1.48 ± 0.17 vs. 2.59 ± 0.18; *p* < 0.001) was documented in children receiving supervised NSI compared to those not instructed for NSI performance.

**Conclusions:** These data suggest that NSI should be considered in the therapeutic management of children with RAOM, and should be routinely prescribed as a daily adjunctive treatment to reduce acute infectious exacerbations in otitis-prone patients. Accurate parents training is crucial in order to improve children compliance and treatment effectiveness.

## Introduction

Acute otitis media (AOM) is the most frequent bacterial disease affecting infants and young children (1) as almost all children have experienced at least one episode by the age of 3 years, and about one-third have experienced three or more episodes (2). In some children, the so-called otitis-prone children, middle ear infections may recur, and in case of at least three documented and distinct AOM episodes in a period of 6 months, or at least four in a period of 12 months (3) we can speak about recurrent acute otitis media (RAOM). Otitis-prone children are predisposed to long-term functional sequalea including chronic otitis media and hearing loss (4), with a global negative medical, social and economic impact on their quality of life. Moreover, dangerous and sometimes life-threating complications may develop, such as otomastoiditis and intra-cranial complications, requiring urgent and aggressive treatments (5).

Recurrent middle ear infections are frequently associated to an impairment in nasopharyngeal homeostasis, mainly sustained by chronic adenoiditis or adenoidal hypertrophy (6) and the related nasopharyngeal bacterial biofilm (7, 8) whose pathogenic role in the development of recurrent or chronic middle ear infections has been largely investigated (7, 8). Beside adenoidal disease and nasopharyngeal bacterial biofilm, several risk factors such as a young age, male gender, little, or no breastfeeding, parental smoking, the use of a pacifier or push-and-pull plastic bottle caps, day-care attendance, allergy, and the presence of an older sibling (9–12) are known to play a causative role in development of chronic or recurrent middle ear inflammation. Therefore, management of otitis-prone children should include all the interventions able to correct (whenever possible) the existing risk factors, including those aimed at improving the patency and clearance of the nasal and nasopharyngeal district.

Nasal saline irrigations (NSI) are largely used as a therapeutic and preventive means in children with allergic rhinitis and upper airway infections such as rhinitis, rhinosinusitis, and adenoiditis (13–15) given their effectiveness in the removal of nasal secretions and in the enhancement of mucociliary clearance (16, 17). However, there is no unanimous consensus about the better formulation, the device, the way of administration and the therapeutic protocols to be used in children (18), and their effective in reducing the number of acute middle ear infections in children with RAOM has not been previously investigated.

The aim of this retrospective study was to evaluate the possible impact of supervised NSI in the reduction of acute middle ear infectious exacerbations in otitis-prone children.

## Materials and Methods

### Study Design and Setting

This retrospective chart review of prospectively recruited otitis-prone children was carried out at Milan University's Department of Clinical Sciences and Community Health and Department of Pathophysiology and Transplantation in August 2018.

The protocol was approved by our local Ethics Committee of Fondazione IRCCS Ca' Granda Ospedale Maggiore Policlinico and was conducted in accordance with the principles of good clinical practice.

### Study Subjects

The study involved the charts of children aged 1–5 years who had attended for first examination our tertiary outpatient clinic of upper respiratory tract infections (CURTI) between January 2015 and January 2018. The children had been referred to the clinic by their general pediatricians who had dealt with their initial episodes of AOM but were unable to manage recurrences effectively.

The children were considered otitis-prone if they had a history of RAOM (19). The episodes of AOM had to be documented by an experienced physician and reported in the children's medical records as any combination of fever, earache, irritability, and hyperemia or opacity accompanied by bulging of the tympanic membrane or otorrhea; at least two episodes had to be supported by otoscopic and tympanometric findings. RAOM was defined as at least three episodes in the preceding 6 months, or at least four episodes in the preceding 12 months (19).

The exclusion criteria were previous nasal treatment with any topical preparation including nasal saline solution regularly administered before first examination at CURTI; nor regular nor adequate NSI administration after first examination at CURTI for parents refusal or reduced children compliance; concomitant systemic diseases; craniofacial, neuromuscular, immunological, syndromic or defined genetic abnormalities; chronic eardrum perforation; previous ear surgery or adenoidectomy; neurosensory hearing loss; immunomodulatory treatment; vitamin D supplementation; the administration of any complementary and alternative medicines.

### Interventions

The retrospective chart review considered the children's demographic data and clinical history. At first examination, all the known risk factors for development of recurrent middle ear disease (including a personal or family history of allergy, little or no breast-feeding, the use of a pacifier or push-and-pull plastic bottle caps, parental smoking, pneumococcal conjugate and influenza vaccination, adenoidal disease, day-care attendance, and the presence and number of older siblings) (9–12) were investigated and, whenever possible, corrected.

Daily nasal irrigations with 0.9% saline solution were prescribed to all the patients (twice a day administration by means of a bulb syringe of 10 and 20 ml per nostril, respectively in children aged 1–3 years and in those aged more than 3 years) (18).

Since January 2016, during the first examination we started to train the parents to supervised NSI by means of detailed verbal instructions about the position and performing modality as it follows: infants should be lying down on their side, whereas older children bend forward over a sink with their head tilted down and a little to a side in the older ones. After washing his\her hands before each use, the care giver should fill up the syringe with warm saline solution taken from a pierced rubber bottle cap, then remove the needle and proceed with gentle and slow nasal irrigation with the syringe directed toward the ipsilateral ear.

A practical demonstration was also performed by the clinician, and the parents received a brochure with written instructions summarizing the main steps and contrivances to be followed to correctly perform NSI.

In this group of patients, a practical demonstration was required to the parents during the second examination 4 months later, in order to assure the compliance to treatment.

At a 4-months follow-up visit, the number of documented episodes of AOM, upper respiratory tract infections and antibiotic treatment was recorded.

### Statistical Analysis

The sample size was computed considering published data regarding the prevalence of recurrent acute otitis media episodes in otitis-prone children (20); assuming a standard deviation of 0.15, it was calculated that 42 subjects for each group would lead to a beta error margin of 0.20, an alpha value of 0.05, and a power of 80%.

The statistical analysis was mainly designed to detect any possible difference in the number of infectious recurrences between otitis-prone children not instructed to NSI performance and those receiving supervised NSI.

The results are given as absolute numbers and percentages, or arithmetical mean values ± standard deviation. Dichotomous outcomes were analyzed using contingency table analysis and Chi squared test, and continuous variables using Wilcoxon-Mann-Whitney test. Univariate and multivariate logistic regression were used to test the effect of possible confounders (i.e., age, gender, allergy, adenoidal disease). The data were analyzed using STATA 10.0 software (StataCorp, College Station, TX, USA); a *p* < 0.05 was considered statistically significant.

## Results

The final analysis was based on the data contained in 173 charts relating to 99 males (57.3%) and 74 females (42.8%) with a mean age of 30.9 ± 7.3 months. Ninety children (52.0%) had not been instructed to perform NSI, while the remaining 83 (48.0%) patients had received supervised NSI.

The two study groups we comparable at the baseline for demographic and clinical variables, including risk factors for RAOM (Table 1).

**Table 1:** Demographic and clinical characteristics of the study groups at the baseline.

**Characteristics**	**Children not instructed to NSI performance (No. = 90)**	**Children instructed to supervised NSI (No. = 83)**	***p*-value**
Mean age ± SD, months	26.94 ± 1.15	31.46 ± 2.16	n.s.
Males	53 (58.9%)	46 (55.4%)	n.s.
Mean No. of AOMs in previous 12 months ± SD	4.18 ± 0.16	4.12 ± 0.23	n.s.
Allergy (%)	6 (11.8%)	17 (20.5%)	n.s.
Little or no breastfeeding (%)	20 (22.2%)	18 (21.7 %)	n.s.
Adenoidal disease	23 (25.5%)	19 822.9%)	n.s.
Day-care attendance (%)	73 (81.1%)	75 (90.4%)	n.s.
Use of pacifier (%)	30 (33.3%)	25 (30.1%)	n.s.
Parental smoking (%)	24 (26.7%)	26 (31.3%)	n.s.
Presence of older siblings (%)	45 (50.0%)	38 (45.8%)	n.s.

*NSI, nasal saline irrigations; No., number; SD, standard deviation; AOM, acute otitis media; n.s., not significant*.

At the follow-up visit a significant reduced number of AOM episodes (1.03 ± 0.14 vs. 2.08 ± 0.16; *p* < 0.001) as well as antibiotic treatments (1.48 ± 0.17 vs. 2.59 ± 0.18; *p* < 0.001) was documented in children receiving supervised NSI compared to those not instructed for NSI performance (Table 2).

**Table 2:** Clinical outcomes of the study groups.

**Outcomes**	**Children not instructed to NSI performance (No. = 90)**	**Children instructed to supervised NSI (No. = 83)**	***p*-value**
Mean No. of all AOMs in the following 4 months ± SD	2.07 ± 0.16	1.03 ± 0.14	< 0.001
Mean No. of AOMs with STMP in the following 4 months ± SD	1.32 ± 0.16	0.66 ± 0.11	< 0.001
Mean No. of AOMs without STMP in the following 4 months ± SD	0.75 ± 0.12	0.47 ± 0.11	n.s.
Mean No. of antibiotic treatments in the following 4 months ± SD	2.59 ± 0.18	1.49 ± 0.17	< 0.001
Mean No. of URTI in the following 4 months ± SD	1.58 ± 0.13	1.32 ± 0.17	n.s.

*NSI, nasal saline irrigations; No., number; SD, standard deviation; AOM, acute otitis media; n.s., not significant; STMP, spontaneous tympanic membrane perforation; URTI, upper respiratory tract infections*.

No significant relationship was found between confounders (i.e., age, gender, allergy, and adenoidal disease) and study groups at univariate logistic regression analysis (Table 3), and all the variables tested resulted to be not confounders on clinical outcomes at multivariate logistic regression analysis (Table 4).

**Table 3:** Results of univariate logistic regression analysis.

**Variables**	***p*-value**
Gender	0.34
Age	0.38
Allergy	0.14
Adenoidal disease	0.24

**Table 4:** Results of multivariate logistic regression analysis.

**Variables**	***p*-value**
Gender	0.11
Age	0.09
Allergy	0.21
Adenoidal disease	0.33
AOM episodes in the following 4 months	<0.001
AOM episodes with STMP in the following 4 months	0.002
Antibiotic treatments in the following 4 months	0.018

*AOM, acute otitis media; n.s., not significant; STMP, spontaneous tympanic membrane perforation*.

## Discussion

NSI have been used for centuries to remove nasal secretions and facilitate nasal drainage (16, 17) and still today are widely prescribed by pediatricians as a simple, inexpensive and well tolerated way to routinely clean the upper airways (18). They are generally considered safe, as despite colonization of nasal saline solution used for NSI by cutaneous and environmental germ is possible, respiratory pathogens are usually not involved and there is no evidence that this condition would facilitate the development of any related infection (21).

NSI proved to be effective in the treatment of children with upper respiratory tract infections and in controlling the symptoms of allergic rhinitis, thus reducing the need for oral antihistamine or intranasal steroid therapy administration (15) and their use has been included in the treatment guidelines for selected respiratory diseases by some scientific societies (12).

Some studies previously documented the positive impact of medicated NSI (especially when administered by means of micronized nasal douches) in the treatment of children with chronic adenoiditis associated with recurrent or chronic middle ear infections (22, 23). In particular, Torretta et al. (22, 23) reported the clinical beneficial effect of a topical nasal sodium hyaluronate solution in children with recurrent or chronic middle ear inflammations associated with chronic adenoiditis (22, 23). However, the possible effect of supervised daily nasal irrigations with 0.9% saline solution performed by means of a bulb syringe in children with RAOM has not been previously investigated. This is the first study evaluating the role of NSI in preventing acute middle ear infections in otitis-prone children: our results documented that children receiving supervised NSI developed a significantly reduced number of AOM episodes compared to those not instructed to correct NSI performance in the 3-months period after recruitment. In particular, in children belonging to the control group the number of both AOM episodes as a whole and AOM episodes with spontaneous tympanic membrane perforation was about twice that which experienced in children belonging to the study group. Moreover, the number of antibiotic treatments during the follow-up period was significantly reduced in children receiving supervised NSI compared to controls.

The presumed mechanism of action probably consists in removing nasal secretions and the antigens and inflammatory mediators such as histamine and prostaglandins here accumulated, with the following improvement in ciliary beat frequency and mucus clearance (15, 16).

Possible limitations of the present study lie in its retrospective nature, in the reduced follow-up period, and in the lack or randomization which may partially bias our results; therefore, prospective controlled trials would be welcome to confirm the impact of supervised NSI in modification of the natural history of RAOM.

In conclusion, our results create rational for prospective controlled trials that should be dedicated to test the possible impact of supervised NSI on natural history of disease in otitis-prone children. If further studies will confirm the positive effect of supervised NSIs, they should be considered in the future in the therapeutic management of children with RAOM, possibly being routinely prescribed as a daily adjunctive treatment to reduce acute infectious exacerbations in otitis-prone patients. In addition, given that their effective could be influenced by the way of administration, further studies should be test the impact of different therapeutic protocols and way of administration, too. However, given that various dosing schedules and types of solution and device are available, and no standardized therapeutic protocols are homogeneously used, accurate parents training with both practical demonstrations and written detailed instructions should be desirable in order to improve not only children tolerability and compliance, but also treatment effectiveness.

## Author Contributions

ST: drafted the manuscript. PM: helped in drafting the manuscript. FF and MF: performed data extraction. TI: performed literature search. LP: made important intellectual contributions to the paper.

### Conflict of Interest Statement

The authors declare that the research was conducted in the absence of any commercial or financial relationships that could be construed as a potential conflict of interest.

## References

[1] Paradise JL Rockette HE Colborn DK Bernard BS Smith CG Kurs-Lasky M.. Otitis media in 2253 Pittsburgh-area infants: prevalence and risk factors during the first two years of life. Pediatrics. (1997) 99:318–33. 10.1542/peds.99.3.318 9041282

[2] Casselbrant ML Mandel EM Epidemiology. In: Rosenfeld RM Bluestone CD, editors Evidence-Based Otitis Media. 2nd ed. London: BC Decker (2003). p. 147–62.

[3] Morris MC Almudevar AL Casey JR Pichichero ME. Familial and microbiological contribution to the otitis-prone condition. Int J Pediatr Otorhinolaryngol. (2015) 79:2174–7. 10.1016/j.ijporl.2015.09.043 26490785 PMC4972179

[4] Cordeiro FP da Costa Monsanto R Kasemodel ALP de Almeida Gondra L de Oliveira Penido N. Extended high-frequency hearing loss following the first episode of otitis media. Laryngoscope. (2018) 128:2879–84. 10.1002/lary.27309 30194735

[5] Ren Y Sethi RKV Stankovic KM. acute otitis media and associated complications in United States emergency departments. Otol Neurotol. (2018) 39:1005–11. 10.1097/MAO.0000000000001929 30113560 PMC6097248

[6] Marseglia GL Poddighe D Caimmi D Marseglia A Caimmi S Ciprandi G. Role of adenoids and adenoiditis in children with allergy and otitis media. Curr Allergy Asthma Rep. (2009) 9:460–4. 10.1007/s11882-009-0068-4 19814919

[7] Torretta S Drago L Marchisio P Gaffuri M Clemente IA Pignataro L Topographic distribution of biofilm-producing bacteria in adenoid subsites of children with chronic or recurrent middle ear infections. Ann Otol Rhinol Laryngol. (2013) 122:109–13. 10.1177/000348941312200206 23534125

[8] Torretta S Marchisio P Drago L Baggi E De Vecchi E Garavello W. Nasopharyngeal biofilm-producing otopathogens in children with nonsevere recurrent acute otitis media. Otolaryngol Head Neck Surg. (2012) 146:991–6. 10.1177/0194599812438169 22357644

[9] Marchisio P Nazzari E Torretta S Esposito S Principi N. Medical prevention of recurrent acute otitis media: an updated overview. Exp Rev Anti Infect Ther. (2014) 12:611–20. 10.1586/14787210.2014.899902 24678887

[10] Marom T Marchisio P Tamir SO Torretta S Gavriel H Esposito S Complementary and alternative medicine treatment options for otitis media: a systematic review. Med (Baltimore). (2016) 5:e2695 10.1097/MD.0000000000002695 PMC4753897 26871802

[11] Torretta S Marchisio P Cappadona M Baggi E Pignataro L. Habitual use of push and pull plastic bottle caps is more prevalent among children with recurrent acute otitis media. Int J Pediatr Otorhinolaryngol. (2013) 77:1179–82. 10.1016/j.ijporl.2013.04.032 23726954

[12] Uhari M Mäntysaari K Niemelä M. A meta-analytic review of the risk factors for acute otitis media. Clin Infect Dis. (1996) 22:1079–83. 10.1093/clinids/22.6.1079 8783714

[13] Wald ER Applegate KE Bordley C Darrow DH Glode MP Marcy SM. Clinical practice guideline for the diagnosis and management of acute bacterial sinusitis in children aged 1 to 18 years. Pediatrics. (2013) 132:e262–80. 10.1542/peds.2013-1071 23796742

[14] Casale M Moffa A Cassano M Carinci F Lopez MA Trecca EMC. Saline nasal irrigations for chronic rhinosinusitis: from everyday practice to evidence-based medicine. an update. Int J Immunopathol Pharmacol. (2018) 32:2058738418802676. 10.1177/2058738418802676 30350744 PMC6201180

[15] Marchisio P Varricchio A Baggi E Bianchini S Capasso ME Torretta S. Hypertonic saline is more effective than normal saline in seasonal allergic rhinitis in children. Int J Immunopathol Pharmacol. (2012) 25:721–30. 10.1177/039463201202500318 23058022

[16] Khianey R Oppenheimer J. Is nasal saline irrigation all it is cracked up to be? Ann Allergy Asthma Immunol. (2012) 109:20–8. 10.1016/j.anai.2012.04.019 22727153

[17] Talbot AR Herr TM Parsons DS. Mucociliary clearance and buffered hypertonic saline solution. Laryngoscope. (1997) 107:500–3. 10.1097/00005537-199704000-00013 9111380

[18] Marchisio P Picca M Torretta S Baggi E Pasinato A Bianchini S. Nasal saline irrigation in preschool children: a survey of attitudes and prescribing habits of primary care pediatricians working in northern Italy. Ital J Pediatr. (2014) 40:7. 10.1186/1824-7288-40-47 24887239 PMC4041066

[19] Gates GA Klein JO Lim DJ Mogi G Ogra PL Pararella MM. Recent advances in otitis media. 1. definitions, terminology, and classification of otitis media. Ann Otol Rhinol Laryngol. (2002) 188:8–18. 10.1177/00034894021110S304 11968863

[20] Rosenfeld RM Kay D. Natural history of untreated otitis media. Laryngoscope. (2003) 113:1645–57. 10.1097/00005537-200310000-00004 14520089

[21] Torretta S Mattina R Talloru F Sala G Cornelli S Bezze E. Bacterial contamination of saline nasal irrigations in children: an original research. Am J Infect Control. (2018) 47:95–7. 10.1016/j.ajic.2018.06.012 30201415

[22] Torretta S Marchisio P Rinaldi V Carioli D Nazzari E Pignataro L. Endoscopic and clinical benefits of hyaluronic acid in children with chronic adenoiditis and middle ear disease. Eur Arch Otorhinolaryngol. (2017) 274:1423–9. 10.1007/s00405-016-4327-4 27695944

[23] Torretta S Marchisio P Rinaldi V Gaffuri M Pascariello C Drago L Topical administration of hyaluronic acid in children with recurrent or chronic middle ear inflammations. Int J Immunopathol Pharmacol. (2016) 2:438–42. 10.1177/0394632016656012 PMC5806751 27481884

